# Outcome of Jejuno-Ileal Atresia Associated with Intraoperative Finding of Volvulus of Small Bowel 

**Published:** 2012-07-01

**Authors:** Shalini Sinha, Yogesh Kumar Sarin

**Affiliations:** Department of Pediatric Surgery, Maulana Azad Medical College, New Delhi-110002

**Keywords:** Jejunoileal atresia, volvulus, leakage, outcome

## Abstract

Aim: To compare the outcome of patients with jeuno-ileal atresia (JIA) associated with the intraoperative finding of volvulus of small bowel (group A) with that of JIA without volvulus (group B).

Materials and Methods: It is a retrospective observational study conducted at one of the two units of Pediatric Surgery, in a tertiary care public hospital of India, from January 2001 to December 2010. Hospital records were retrieved and analyzed. During this time period, 65 patients with JIA were operated of which 40 (61.5%) had ileal atresia (IA) and 25 (38.5%) had jejunal atresia (JA). Eleven (16.9%) patients had associated intraoperative finding of volvulus of small bowel (Group A) and were studied and compared with group B- not associated with intraoperative findings of volvulus of small bowel (n=54). The demography, clinical features, operative findings, associated anomalies, anastomotic leakage, and outcome were compared.

Results: Group A comprising of 6 boys and 5 girls, had 8 IA and 3 JA; one case each of Type 3b and Type 4 JIA was seen. Associated anomalies included meconium ileus (n=2), Down’s syndrome (n=1) and malrotation (n=1). Anastomotic leak rate was 75% for IA and 66.7% for JA. The mortality was 91% in Group A, 100% for IA and 67% for JA. Group B comprising of 37 boys and 17 girls, had 32 IA and 22 JA; 2 cases of Type 4 and 1 case of Type 3b JIA was seen. Associated anomalies were malrotation (n=2), meconium ileus (n=1), exomphalos (n=1), gastroschisis (n=1) and ileal duplication cyst (n=1). The anastomotic leak rate for JA was 8/21 (38.1%) and IA was 3/28 (10.7%); persistent obstruction was seen in 3/21(14.3%) JA and 1/28 (3.6%) IA patients. In group B, overall mortality rate was 8/22 (36.4%) for JA and 9/32 (28%) for IA. The morbidity and mortality was significantly higher in group A when compared to group B.

Conclusions: JIA associated with volvulus (without malrotation) is a sinister entity with a dismal outcome in our experience.

## INTRODUCTION

Jejunoileal atresias (JIA) account for about one third of cases of all neonatal intestinal obstructions [1]. It is well established that a late intrauterine mesenteric vascular accident, such as, in-utero intussusception [2, 3], intestinal perforation, segmental volvulus [3], malrotation, internal hernia or thromboembolism; constitutes the etio-pathology of this anomaly. It has also been seen in association with omphalocele, gastroschisis and meconium ileus [4]. In addition, maternal intake of vasoactive drugs like ephedrine and acetaminophen, substance abuse with cocaine and smoking have been shown to cause multiple JIA [5]. The incidence of volvulus causing JIA has been reported as about 27% [3,6]. In our experience, JIA associated with volvulus was encountered less frequently and these patients had a more dismal outcome. Hence, we decided to deliberate further on this group. It is understood that JIA could be secondary to volvulus or the volvulus could be secondary to JIA. Hence, the index study included all those patients who had intraoperative findings of small bowel volvulus regardless of the etiology of JIA.

## MATERIALS AND METHODS

A retrospective analysis of all neonates who were operated for intestinal atresia in one of the two units of Pediatric Surgery, in a tertiary care public hospital of a resource challenged country over a decade (January 2001 to December 2010) was done by retrieval of hospital records. There were 65 patients with JIA during the stipulated time period of which 40 (61.5%) had ileal atresia (IA) and 25 (38.5%) had jejunal atresia (JA). Of the 65 patients, 11 (16.9%) were found to have associated volvulus intra-operatively (Group A). We studied them in detail with respect to their antenatal diagnosis, clinical features, associated anomalies, operative procedures performed, complications and outcome. The complications and outcome of group A (n=11) were compared with group B, namely, JIA without associated volvulus (n=54). The statistical analysis was done using the Fisher’s Exact Test since the sample size was small.

## RESULTS

Group A

Antenatal: Prenatal ultrasound was done in 3 mothers of which only 1 third trimester scan detected polyhydromnios with dilated bowel loops. Even though the scan findings were highly suggestive of JIA, the neonate was not referred antepartum to a tertiary care centre like ours. There was history of maternal diarrhea in 1 mother during the third trimester of pregnancy. No other antenatal risk factors could be identified.

Demography: Six boys and 5 girls with a mean age of 2.5 days were found to have JIA (8 IA and 3 JA) associated with intraoperative findings of volvulus. The mean birth weight was 2539 g (range 1700 – 3500g). The mean birth weight for JA (2333g) was comparable to that of IA (2610g). The gestational age was >42 weeks (n=1), 37 – 42 weeks (n= 7) and 32 -36 weeks (n=3). None had any features of birth asphyxia.

Clinical Presentation: All the neonates presented with symptoms of bilious vomiting and abdominal distension after birth. Failure to pass meconium was seen in 5/11 newborns. A 5-days-old baby presented with perforation. Another one came at 31 hours of life in shock with gangrene. Though the median age at presentation was 2 days (range 1 – 7 days), about one fourth (3/11) of these patients were referred late, i.e., after 2nd day of life.

Associated anomalies: Table 1 illustrates the associated anomalies. Malrotation was seen in only 1 patient. One neonate with meconium ileus was also suspected to have tetanus neonatorum. 

**Figure:**
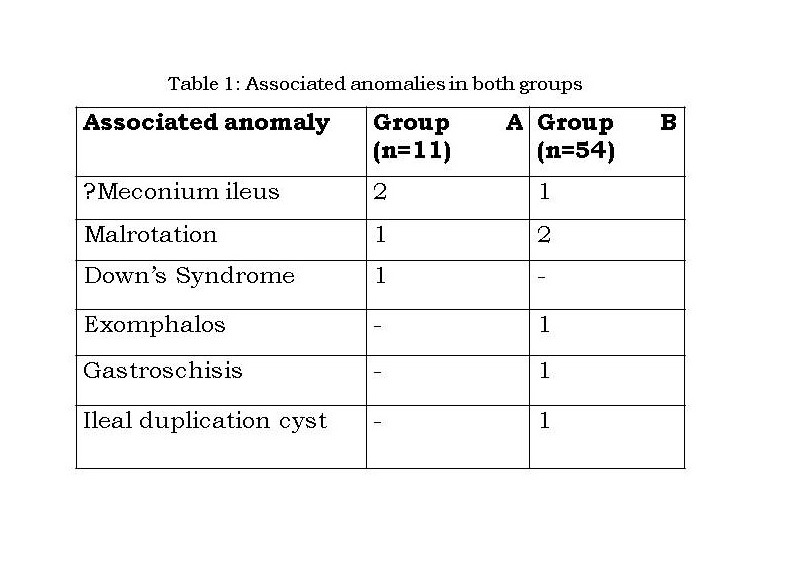
Table 1: Associated anomalies in both groups

Diagnosis: X-ray abdomen showed multiple air-fluid levels with distal cut-off in all patients and free intra-peritoneal air in one. Preoperative contrast study was not required in any of the patients.

Operation: After preoperative stabilization of fluid, acid base and electrolyte balance, the patients were taken up for exploratory laparotomy. The associated volvulus was seen involving the proximal segment in 5 patients, the distal segment in 4 patients and sequestered between the proximal and distal segments in 2 patients. Dense adhesions were present in all of them. Although pre-operative X-ray showed free air in only 1 patient, intra-operatively, 2 patients had perforation. Gangrenous necrosis was found in 3 more newborns and type 3b Apple Peel anomaly was seen in 1 patient. In addition to the volvulus, one neonate also had bands and 3 atresias (2 membranous and 1 complete) over a length of 5cm in the mid ileum with a proximal sealed off antenatal perforation as evidenced by dense adhesions and meconium specs in the peritoneal cavity. The length of remaining bowel was adequate in all except 3 patients - 40cm of small bowel with ileocaecal valve (n=2) and only 15 cms of jejunum without ileocaecal valve (n=1). Among the 3 JA with volvulus, 2 had end to back anastomosis and 1 baby with malrotation and resorption of almost entire small bowel underwent jejunocolic anastomosis with tapering of jejunum. Of the 8 IA with volvulus, 4 underwent end-to-back anastomosis, 2 had Bishop Koop’s stoma due to features of meconium ileus and the remaining 2 with perforation peritonitis underwent divided ileostomy.

Complications: Two out of three primary anastomoses in JA with associated volvulus leaked (66.6%) and 1 had persistent bilious aspirates despite a normal dye study. He developed septicemia and the parents took the baby home against medical advice on the 12th postoperative day. Three out of 4 primary anastomoses in IA with volvulus leaked fatally (75%) and 1 patient died within 48 hours of surgery with refractory shock and coagulopathy. The second patient with divided ileostomy also required multiple reoperations for fresh gangrenous areas and bile leak from main wound. He finally succumbed to sepsis on day 27 of life. Both the patients who underwent Bishop Koop’s stoma died of sepsis and meningitis within 48 hours of surgery.

Outcome and follow up: The mortality in neonates with JIA was 91%; 100% for IA and 67% for JA. Only 1/8 patients with IA associated with volvulus survived beyond the 1st month of life. This baby had undergone primary diversion due to gangrene and perforation. He was taken up for ileostomy closure at 38 days of age. During the surgery for internal jugular central venous access, he developed bradycardia due to vagal stimulation and died the same evening. Of the entire group A, only one patient with JA having associated Down’s syndrome survived after multiple surgeries for anastomotic leak. She was followed up for 18 months when she developed adhesive intestinal obstruction requiring another surgical intervention.

Group B

The median age of the remaining 54 patients with JIA without volvulus was 3 days (range 1-17 days); 32 (59.3%) had IA and 22 (40.7%) had JA. The demographic details of this group are enumerated in Table 2 and were more or less similar to those of group A, hence eliminating any confounding factors. Associated anomalies are mentioned in Table 1. Of the 22 JA, all except 1 had primary anastomosis. This child with complicated meconium ileus and perforation-peritonitis required divided jejunostomy which was followed by an early closure; and he survived. The anastomotic leak rate was 8/21 (38.1%), persistent obstruction was seen in 3/21(14.3%) and mortality rate was 8/22 (36.4%). Two patients died within 48 hours of surgery, 1 of which had an intraoperative anesthetic complication. One patient died 1 month after surgery due to aspiration. The remaining 6 died either of sepsis or anastomotic leak. Twenty eight IA had primary anastomosis of which 3 (10.7%) leaked and 1 (3.6%) had persistent obstruction. Four patients underwent primary diversion due to presence of perforation-peritonitis and gangrene. The mortality was 9/32 (28%). Two of the 9 deaths were unrelated to surgery (aspiration and delayed sepsis). Three patients died within 48 hours of surgery, 3 died within 10 days of surgery due to leak and 1 patient died of sepsis following ileostomy closure.

**Figure:**
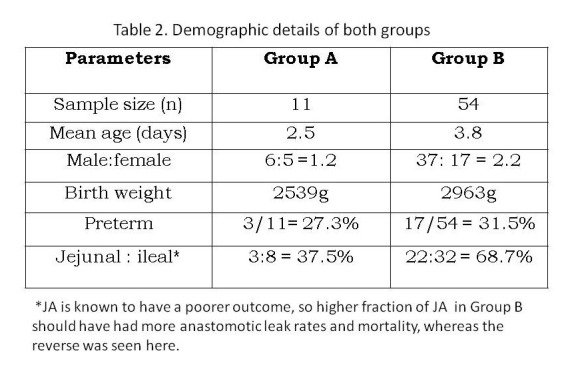
Table 2: Demographic details of both groups

Statistical analysis

The overall anastomotic leak rate for neonates who underwent primary anastomosis was significantly higher in group A as compared to group B (p = 0.0161). Similarly, the overall mortality was significantly higher in group A (p = 0.0004). The analysis is summarized in Table 3. On comparing the outcome between JA and IA within each group, there was no significant difference found in group A with respect to anastomotic leak (p = 1.000) or mortality (p = 0.2727). However, in group B, though the mortality rates were not significantly different between JA and IA (p = 0.5625); the anastomotic leak rates were significantly higher in JA (p = 0.0372).

**Figure:**
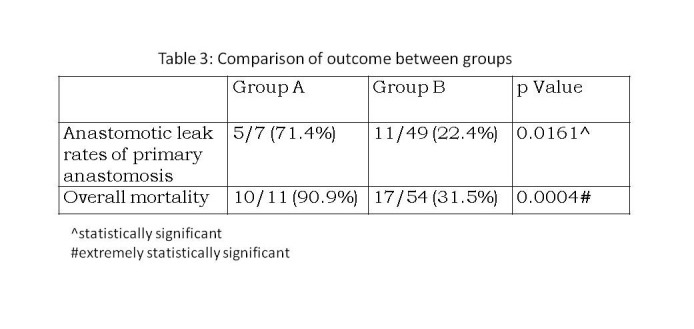
Table 3: Comparison of outcome between groups

## DISCUSSION

Various authors have described JIA associated with volvulus [3,6-8]. The incidence of JIA with volvulus in this study was16.9 % which is much lower than that reported in literature [3,6]. Komuro et al reported 13 cases of JIA complicated with volvulus (27%) in a series of total 48 patients. They mention three types of volvulus- type 1, involving the proximal end as opposed to the distal end in 7 cases; type 2, involving only the distal end in 5 cases; and type 3, located between the proximal and distal blind end in 1 case [3]. Going by this classification, majority of our patients had type 1 or 2 volvulus. They suggested that not only does intra-uterine volvulus cause JIA secondary to ischemic necrosis of bowel; the reverse may also be true. The hugely dilated blind ending proximal bowel along with contraction of the mesenteric defect and increased peristalsis of the proximal bowel may lead to volvulus [3]. The association with complicated meconium ileus is easy to comprehend wherein the dilated ileum loaded with viscid meconium may develop volvulus followed by bowel ischemia and JIA [3]. We found the incidence of meconium ileus to be much higher in JIA associated with volvulus (2/11 vs 1/54). Stollman et al reported the incidence of cystic fibrosis with JIA as 9% and recommend screening of all JIA neonates to rule out cystic fibrosis [8]. As has been reported by several authors [3, 8], malrotation with midgut volvulus was rare in our series (1/11). This can be explained by the fact that intra-uterine midgut volvulus usually leads to fetal demise [9]. Black et al postulated basilar and segmental mesenteric defects as the primary etiology for JIA as it unifies all the lesions observed in JIA under one theory [10]. Other than meconium ileus, omphalo-mesenteric bands [11] and mesenteric defects causing obstruction and/ or subsequent twisting of bowel, the exact pathogenesis of intrauterine volvulus without malrotation is unclear [10, 12,13,14].

Midgut volvulus due to malrotation can be suspected on prenatal ultrasound by demonstration of the “Whirlpool” or “snail sign” on color doppler [12]. It is possible to diagnose JIA associated with volvulus prenatally though most of these cases also have malrotation [17]. Antepartum MRI can be a useful tool, to delineate with accuracy, the features of both JIA and volvulus [18]. However in our set-up, prenatal diagnosis of fetal anomalies is still in its primitive stage, and in all the 11 cases the presence of volvulus was detected only during surgery. Besides, the referral systems are poorly organized as evidenced by failure of timely referral to a tertiary care centre in the only prenatally diagnosed case of JIA with volvulus.

Majority of our cases were surgically managed by end-to-back anastomosis after resection of atretic ends (86%) and only 9/65 required diversion at the time of initial operation. However, due to presence of dense adhesions, gangrene and perforation, half of the IA with volvulus (4/8) underwent a stoma. In the 70s, ostomy was done for almost 50% of JIA [19]. Off late, owing to the high occurrence of complications, more surgeons tend to avoid stomas in neonates [20, 21]. Hence in a recent report by Stollman in 2009, temporary ostomy was required for only 26% cases of JIA [8]. The need for tapering enteroplasty in JIA has been reported as 24% [19]. In our series, 1/11 in group A and 3/54 in group B (2 JA and 1 IA), underwent bowel tapering. Two of these tapering procedures were done at the time of subsequent surgeries for anastomotic leak or persistent functional obstruction. 

The presence of bowel complications like intestinal atresia, perforation, necrosis or volvulus stratifies gastroschisis as ‘complex’ and these cases have been shown to have a poorer outcome when compared to simple gastroschisis [23, 24]. Prasad et al reported an increase in mortality in JIA when there were multiple atresias (57%), apple peel atresia (71%), and when JIA was associated with meconium ileus (65%), meconium peritonitis (50%) and gastroschisis (66%) [25]. There are other reports from developing countries stating 41% mortality rates for JIA which are comparable to our results [26].

Our observation that the mortality (90.1% vs 31.5%) and morbidity (71.4% vs 22.4%) was significantly higher in the JIA associated with volvulus when compared to JIA without volvulus was proved to be statistically significant. A similar comparison of outcome could not be found in literature as most of the series mention overall mortality and morbidity rates. Contrary to our observation, a study of 10 neonates with prenatal intestinal volvulus by Raherison et al in 2012, showed a better outcome with 90% survival [27]. There were 2 cases of JIA in this series and both did well. However the number of cases with JIA and volvulus was small in this series.

## Conclusion

JIA associated with intraoperative findings of volvulus is a sinister entity with a dismal outcome as proved in this study. Both the anastomotic leakage and mortality rate were significantly higher in patients of JIA associated with intraoperative findings of small bowel volvulus. Further studies are invited to sort out various factors resulting in higher leakage and the resultant mortality in these patients.

## Footnotes

**Source of Support:** None

**Conflict of Interest:** None

